# Analysis of transcription profiles for the identification of master regulators as the key players in glioblastoma

**DOI:** 10.1016/j.csbj.2024.09.022

**Published:** 2024-09-28

**Authors:** Sergey M. Ivanov, Alexey A. Lagunin, Olga A. Tarasova

**Affiliations:** a Department of Bioinformatics, Institute of Biomedical Chemistry, Pogodinskaya Street, 10 bldg. 8, Moscow 119121, Russia; b Department of Bioinformatics, Pirogov Russian National Research Medical University, Ostrovityanova Street, 1, Moscow 117997, Russia

**Keywords:** Glioblastoma, Individual samples, Gene expression, Transcription factors, Master regulators, Feedback loops

## Abstract

Glioblastoma (GBM) is the most common malignant brain tumor with poor overall survival. Current treatment management for GBM has low efficacy, mainly due to high inter-patient heterogeneity. The transcription profiles in GBM define cell properties essential for tumor progression. We have developed an approach for the identification of master regulators (MRs) that are responsible for the gene expression changes in GBM. The approach is based on transcription factor enrichment analysis with subsequent “upstream” analysis in the signaling network. The main feature of the approach is that all calculations are performed for transcription profiles from individual samples, which allows taking into account GBM transcription heterogeneity. We identified 451 MRs that were up-regulated or down-regulated and, thus, were important parts of positive feedback loops. The number of MRs in the samples correlated with the degree of tumor immune infiltration, while the differences in MR profiles were generally consistent with the known GBM subtypes: mesenchymal, classical, and proneural. MRs densely interact with each other in the signaling network that may be associated with the robustness to pharmacological intervention. We identified 102 receptors among MRs, which is coherent with the importance of cell-cell interactions for GBM progression. The role of some of them in GBM is not currently investigated: lysophosphatidic acid receptors 5 and 6, sphingosine-1-phosphate receptor 4, lysophosphatidylserine receptors GPR34 and GPR174, and G protein-coupled receptors 84 and 132 for fatty acids. Information on the revealed MRs can be used to search for novel therapeutic strategies to treat GBM.

## Highlights

• The master regulators regulating gene expression in glioblastoma were identified.
• The 451 master regulators are important components of positive feedback loops.
• Differences in master regulators profiles are associated with glioblastoma subtypes.
• Master regulators form a dense regulatory network, sustainable to pharmacological intervention.
• Lysophosphatidylserine and fatty acid receptors may be essential for glioblastoma progression.

## Graphical Abstract

**Figure:**
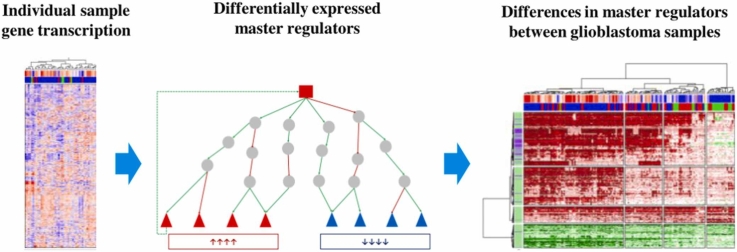

## 1 Introduction

Glioblastoma (GBM) is the most common and deadliest malignant brain tumor with a 5-year survival rate of only 7 %. Although overall survival for malignant brain tumors increased in the last decade, the survival rate for GBM remains low [1]. Current treatment management for GBM is based on surgery followed by radiotherapy, chemotherapy, and immunotherapy [2], [3], [4], [5], [6]. Despite the existence of various anti-cancer drugs containing kinase inhibitors [7], they are not used for GBM treatment because of the low blood-brain barrier permeability [8], side effects, and toxicities [9], [10], [11] as well as the resistance to chemotherapy [10]. Another important obstacle to effectively curing GBM is its high inter-patient and intra-tumor heterogeneity. Therefore, estimating heterogeneity may help to select the most effective drug combinations and develop novel therapeutic strategies for GBM treatment. To estimate GBM heterogeneity, various OMICs technologies are applied. To date, a large amount of cancer-related data is generated and publicly available in various databases. For instance, The Cancer Genome Atlas (TCGA) is the largest repository of genomic, epigenomic, transcriptomic, and proteomic data on tumors, including GBM [12], [13]. The most commonly used type of OMICs data is transcriptomics obtained by bulk and single cell RNA sequencing technologies. The results of bulk sequencing can be used for the comparison of gene transcription profiles from biopsy samples between different patients. Previously, cluster analysis of such transcription profiles allowed revealing at least three GBM transcription subtypes: classical (CL), mesenchymal (MES), and proneural (PN) [14], [15]. The analysis of genomics and clinical data associated with the same samples made it possible to estimate the molecular characteristics of the revealed subtypes. They are characterized by different mutation profiles, mutated genes, and chromosome alterations as well as by the differences in tumor microenvironment and survival times. For instance, the CL subtype is associated with chromosome 7 amplification paired with chromosome 10 loss. At the gene level, the CL subtype is characterized by an increase in the expression of EGFR and NES genes, Notch (NOTCH3, JAG1, LFNG) and Sonic hedgehog (SMO, GAS1, GLI2) pathways components, and the deletion of CDKN2A gene. The MES subtype is characterized by the deletion of the NF1 gene, increased expression of tumor necrosis factor superfamily and NF-kB pathway genes, and increased mesenchymal markers. It is also associated with higher overall necrosis and inflammatory infiltrates, epithelial-mesenchymal transition changes, and decreased patient survival [15], [16]. The PN subtype is associated with amplification of the locus at 4q12 harboring the PDGFRA gene with its increased expression, a higher rate of mutations in the TP53 and IDH1 genes, younger patient age, and longer survival time compared to other GBM subtypes [15]. In contrast to bulk RNA sequencing that allows comparing gene transcription profiles between hundreds of samples, the single-cell RNA sequencing data is mainly applied to estimate intra-tumor cell heterogeneity in a few samples [1], [17], [18]. The single-cell transcriptomics analysis revealed that malignant cells in GBM exist in four main cellular states: neural progenitor-like (NPC-like), oligodendrocyte-progenitor-like (OPC-like), astrocyte-like (AC-like), and mesenchymal-like (MES-like). The proportions of these types of cells determine a bulk transcription subtype of a sample: the CL and MES subtypes correspond to tumors enriched for the AC-like and MES-like states, respectively; the PN subtype corresponds to the combination of two distinct cellular states, OPC-like and NPC-like [18]. However, in some cases, the proportions of cell types are intermediate, and the corresponding sample cannot be clearly classified to one of three subtypes [15]. Moreover, the corresponding proportions are varied between different regions of the same tumor, and cells of one type are able to transit to another type. The latter property, called “plasticity”, is important for tumor evolution and the development of treatment resistance.

The data on molecular interactions inside and between cells in a tumor is widely used in network analysis of OMICs data [19], [20]. The results of this analysis provide a basis for a holistic view on molecular processes in tumors and allow performing tumor classification, biomarkers identification, and drug repurposing. Most of the previous studies used an analysis of protein-protein interaction networks coupled with the analysis of transcription changes in GBM [21], [22], [23], [24], [25], [26], [27], [28], [29]. The main purpose of this analysis was to identify hubs in networks containing proteins encoded by differentially expressed genes (DEGs). These hubs may represent potential biomarkers and perspective pharmacological targets to treat GBM. Genome-scale metabolic models were also used to model metabolism in GBM cells using steady-state assumption-based methods like flux balance analysis [30], [31], [32]. The simulation of genetic knockouts in metabolic models allowed revealing the potential targets and drugs for GBM treatment. The data on the signaling and gene regulatory networks represented as directed signed graphs were used to search for master regulators (MRs) in GBM [33], [34], [35], [36], [37]. MRs are proteins at the top of the signaling network, which drive cell state transitions, e.g., from healthy to disease states, by induction of gene expression changes [38], [39]. For example, Kalya M. and colleagues compared transcription profiles between samples from patients who survived more than 36 months (long-term survivors) and less than 12 months (short-term survivors) [36]. They identified gene expression changes between two groups and, in turn, transcription factors (TFs) binding sites, which were enriched in the promoters of dysregulated genes including NANOG, NF-κB, REST, FRA-1, and PPARG. Then, the upstream MRs regulating the activity of identified TFs were recognized at the top of the reconstructed signaling network. The genes encoding five MRs were differentially expressed, which means that the revealed MRs are part of positive feedback loops. The identified MRs, particularly insulin-like growth factor binding protein 2 (IGFBP2), vascular endothelial growth factor A (VEGFA), VEGF165, platelet-derived growth factor A (PDGFA), adipocyte enhancer-binding protein (AEBP1), and oncostatin M (OSMR), were proposed as biomarkers and therapeutic targets for enhancing GBM prognosis [36].

Despite the significant impact on the knowledge of molecular mechanisms of GBM development and progression, almost all of the previous studies were focused on globally averaged transcription profiles, ignoring the differences between GBM subtypes, e.g., CL, MES, and PN. Since there are differences in gene transcription between GBM subtypes, this approach may introduce a significant bias into the obtained results. Ideally, transcriptional heterogeneity within the subtypes should also be taken into account. Thus, in the current study, we developed and applied an approach to identify MRs for individual samples, with subsequent analysis of correlations with the data on GBM subtypes and properties of tumor microenvironment. The study included the following stages. First, significant DEGs, TFs, and MRs were identified for individual samples of GBM with the use of publicly available algorithms implemented in various R packages. Second, differentially expressed MRs that are the important parts of positive feedback loops were identified by estimating correlations between the presence of MRs and DEGs in individual samples. Third, pathway enrichment analysis was performed for the identified MRs to estimate the most important pathways in GBM. Fourth, the distribution of the revealed MRs among individual samples, including their relationships with the known GBM subtypes and immune infiltration of tumors, was estimated by the creation of a heatmap with hierarchical clustering of samples and proteins, coupled with deconvolution analysis. Fifth, the creation and analysis of the signaling network containing MRs was performed to estimate the complexity of the gene expression regulation and the potential for therapeutic intervention. Sixth, particular sets of the most important and poorly studied MRs were analyzed and discussed.

As a result, our approach allowed revealing MRs important for GBM development and progression, including many new ones whose role in GBM was described poorly or was not described previously at all. A complex nature of gene expression regulation by the network of interacting MRs was also revealed.

## 2 Materials and methods

### 2.1 Preparation of GBM gene expression profile data

The information about gene transcription in GBM cells measured by bulk RNA sequencing technology was obtained from The Cancer Genome Atlas (TCGA) (https://www.cancer.gov/ccg/research/genome-sequencing/tcga). To download the data on gene expression of protein-coding genes, the TCGAbiolinks R package was used [40]. For further analysis, we selected 155 samples of primary GBM and 5 samples of “healthy” surrounding tissues. We only included protein-coding genes in the analysis because their regulation by transcription factors (TFs) is well described in the literature and databases compared to non-protein-coding genes. After the normalization performed using the TCGAanalyze_Normalization function with the account for GC content and the filtration of genes with the transcription level below a lower quartile, a matrix with data on 160 samples and 14937 genes was obtained.

### 2.2 Estimation of GBM transcriptome subtypes

The information on GBM subtypes previously revealed based on transcription profiles in GBM cells [14], [15] was obtained from TCGA using the GDCprepare function. The information on three GBM subtypes: CL, MES and PN, was available for 133 out of 155 tumor samples. To predict subtypes for the remaining 22 samples, we created a classification model using a Random Forest algorithm implemented in the RandomForest R package with default hyperparameters. The accuracy calculated by the leave-one-out cross-validation procedure was 0.925. After merging the known and predicted data on GBM subtypes, we obtained information about 56 CL, 76 MES, and 23 PN samples.

### 2.3 Identification of differentially expressed genes in individual GBM samples

Differentially expressed genes (DEGs) were identified for each of the 155 GBM samples by comparing a GBM expression profile from the sample with corresponding profiles from 5 samples of “healthy” surrounding tissues. All calculations were performed using methods implemented in the edgeR package. We considered DEGs with a log fold change greater than 1 and less than −1, and p-values less than 0.05 (after Benjamini-Hochberg correction). These thresholds were selected empirically to balance the numbers of DEGs and their statistical significance [38], [39].

### 2.4 Estimation of transcription factor activities from gene expression data

To identify transcription factors (TFs) potentially responsible for the observed gene expression changes, the Virtual Inference of Protein-activity by Enriched Regulon analysis (VIPER) method was used [41]. The information on “TF – gene” regulatory interactions of two types: stimulation and repression, was obtained from the CollecTRI database [42]. The corresponding data was retrieved using the decoupleR package. The analysis was performed for individual samples using the msviper function from the VIPER package and log fold change profiles as input. The obtained normalized enrichment scores (NES) were converted to a binary form as follows: if the NES was positive and the adjusted p-value was less than 0.05, then the TF was considered “active”. Otherwise, if the NES was negative and the adjusted p-value was less than 0.05, the TF was considered “inactive”. The “active” TFs according to CollecTRI data increase (decrease) the expression of genes which are up-regulated (down-regulated). The “inactive” TFs decrease (increase) the expression of genes which are up-regulated (down-regulated). If “inactive” TFs were “active”, they would reverse gene expression profiles to those observed in healthy tissues.

### 2.5 Estimation of upstream master regulators from the inferred transcription factors

To identify upstream master regulators (MRs) in the signaling network that regulate the revealed TFs, we performed a causal network analysis using the CausalR package [43]. The CausalR algorithm, like VIPER, allows identifying two types of MRs: (1) “active” MRs that stimulate “active” TFs and inhibit “inactive” TFs; and (2) “inactive” MRs that stimulate “inactive” TFs and inhibit “active” TFs. The CausalR algorithm calculates the shortest paths between upstream proteins in the signaling network and TFs using information about the sign of edges: activating and inhibiting edges. To perform the analysis, we used the signaling network downloaded from the Omnipath database (https://omnipathdb.org/) via the OmnipathR package. We included into the network those proteins, which were encoded by genes intersected with 14937 genes expressed in GBM (see above). The analysis was performed for each tumor sample, and the obtained data on MRs was presented in a binary form: “active” and “inactive” MRs. For further analysis, we selected MRs with a p-value less than 0.05 calculated by a permutation test. The only hyperparameter value that the user has to consider was “delta” – the length of the shortest paths connecting the upstream proteins in the network with TFs. We sequentially used delta values from 1 to 3 for the identification of MRs and then merged the obtained results. The corresponding lengths of the shortest paths were chosen because the changes in cell signaling and gene expression caused by the modulation of protein function, e.g., by action of a drug on a protein or binding of endogenous ligand with a receptor, decrease with an increase in the distance between this protein and TFs [44], [45], [46]. Thus, the use of shortest paths of long lengths may lead to the identification of many false positive MRs, whereas the use of short length paths may lead to the identification of many false negative MRs. The lengths from 1 to 3 were chosen because these settings allow revealing all known proteins that have well-described important roles in GBM pathogenesis, e.g., EGFR, CDK4, AKT1, BRAF, FOXM1, KRAS, NRAS, NF1, PDGFB, PTEN, STAT3, TP53, VEGFA, and others. The controversial data on MRs, e.g., in cases where MRs were predicted as “active” with some deltas and “inactive” with other deltas, were removed from the final results.

Finally, the obtained data on TFs and upstream MRs was merged for further analysis.

### 2.6 Merging the information on master regulators and differentially expressed genes

We proposed that the most probable and important MRs must regulate their own expression: genes encoding “active” MRs should be up-regulated, whereas genes encoding “inactive” MRs should be down-regulated. Each of the genes encoding MRs was represented by two 155-length vectors with discrete values. The first vector contained the data on MRs: “active” MR – “1”, “inactive” MR – “−1”, MR not identified in a particular sample – “0”. The second vector contained the data on gene differential expression: up-regulation – “1”, down-regulation – “−1”, expression unchanged – “0”. The similarity of two vectors was calculated using a cosine similarity measure with the lsa R package. Bootstrap analysis was used to calculate the 95 % confidence interval for cosine similarity values. MRs with cosine similarity more than 0.3 and a lower limit of the confidence interval greater than 0 were selected for further analysis.

### 2.7 Pathway enrichment analysis

Pathway enrichment analysis was used to identify KEGG pathways (https://www.genome.jp/kegg/pathway.html) associated with “active” and “inactive” MRs. The “enrichR” package was used for this purpose. We selected pathways containing at least two genes encoding MRs and the p-value less than 0.05.

To visualize the obtained pathways, a treemap diagram was created using the “treemap” R package and information on KEGG pathways groups and subgroups (https://www.genome.jp/kegg/pathway.html) for the pathway terms clustering.

Additionally, MRs were classified according to their molecular functions, e.g., receptors, ligands and their subtypes, protein kinases and phosphatases, transcription factors, proteases, etc. The corresponding information was obtained from the PANTHER classification system (https://www.pantherdb.org/) and UniProt database (https://www.uniprot.org/).

### 2.8 Calculation of the immune infiltration scores

To estimate the degree of immune cells infiltration of GBM samples, the ESTIMATE algorithm implemented in the “immunedeconv” R package was used [47], [48]. ESTIMATE is a deconvolution method that uses gene expression profiles to infer the fraction of stromal and immune cells in tumor samples.

To estimate correlation between the presence of MRs (discrete vectors of −1, 0, and 1), and immune infiltration scores, the Kendall correlation coefficient was used. The correlation between expression values of genes encoding MRs and immune infiltration scores was also estimated using the Kendall correlation coefficient. If the coefficients at both discrete and expression levels were positive and significant (p < 0.05), the presence of correlation was considered.

Additionally, we manually classified MRs as immune-specific and not specific, based on the information on the role and expression of corresponding proteins in immune cells from the UniProt database (https://www.uniprot.org/) and literature. Immune-specific MRs include various cytokines and their receptors, components of T-cell receptor, major histocompatibility complex, and some other proteins.

### 2.9 Visualization of the distribution of MRs between GBM samples

To visualize the distribution of MRs between GBM samples, we generated a heatmap with hierarchical clustering of both MRs and samples using the ComplexHeatmap R package. Initially, the discrete vectors containing the data on the MR presence in particular samples (−1, 0, 1 values) and vectors containing the data on the differential expression of MR encoding genes (−1, 0, 1 values) (see above) were merged as follows: The highest score of “3” (“−3”) was considered for MRs that were identified as “actives” (“inactives”) in a given GBM sample, and the corresponding genes were also up-regulated (down-regulated). These MRs were the most probable candidates as regulators of gene expression and were differentially expressed. The other proteins were less probable to be MRs and received the following scores: if the MR was identified as “active” (“inactive”) in a particular GBM sample but the corresponding gene was not up-regulated (down-regulated), it received a score of ”2” (“−2”); if the MR was not identified as “active” (“inactive”) in a given GBM sample but the corresponding gene was up-regulated (down-regulated), it received a score of “1” (“−1”). In other cases, the score was considered to be “0”. The 1 - cosine similarity values between the obtained vectors were used as distances for cluster analysis. Both samples and MRs were clustered based on the obtained values using the Ward method of the hierarchical agglomerative algorithm.

### 2.10 Analysis of master regulator interaction network

We used the Cytoscape platform to visualize the interactions between MRs in the signaling network [49] (https://cytoscape.org/). We performed the controllability analysis to estimate the complexity of the MR network, the most important MRs, and the capacity of therapeutic interventions. The network is controllable if it can be driven from any initial state to any desired final state within some finite time. The analysis was performed using the CytoCtrlAnalyser Cytoscape plugin [50]. We used three algorithms implemented in CytoCtrlAnalyser to perform controllability analysis: the minimum driver node set (MDS) [51], the minimum steering node set (MSS) [52], and the steering nodes for output control [53].

To identify the most important MRs, we calculated outdegree and closeness centrality values for each MR in the network using the CentiScaPe Cytoscape plugin [54]. Outdegree is a number of outcome edges from a particular MR to other MRs in the network. Closeness centrality calculated by CentiScaPe is an inverse sum of lengths of the directed shortest paths from a particular MR to other MRs in the network. The higher the outdegree and centrality values are, the stronger is the influence of MR on other MRs and gene expression.

To identify TFs, which have significant impact on gene expression in GBM samples, we created a separate bulk hierarchical TF-target network. The corresponding data on “TF – gene” regulatory interactions of two types: stimulation and repression, was obtained from the CollecTRI database [42]. To identify the relevant interactions, we used an approach similar to those described in Section 2.6 (see above). All TFs were represented by 155-length vectors with discrete values: “active” TF – “1”, “inactive” TF – “−1”, and TF not identified in a particular sample – “0”. The second group of vectors contained the discrete data on differential expression of TF target genes: up-regulation – “1”, down-regulation – “−1”, and the expression unchanged – “0”. The cosine similarity between the vector for TF and the vector for a target gene represents the potential sign of gene regulation: stimulation and repression. If TF stimulated (repressed) the gene transcription according to CollecTRI data, the cosine similarity value was more than 0.3 (less than −0.3) and the lower limit of the confidence interval was greater than 0 (less than 0), the corresponding TF – gene regulatory interaction was included in the bulk hierarchical TF-target network. The outdegree values for all TFs in the network were calculated using the CentiScaPe plugin. We performed two similar analyses for bulk hierarchical TF-target network: the first analysis took into account all DEGs regulated by TFs, and the second analysis took into account only DEGs encoding MRs.

### 2.11 Single cell RNA sequencing data for the genes encoding master regulators

The single-cell RNA sequencing data for GBM was obtained from the results of the study made by Cyril Neftel and colleagues [18] which is available on the Single Cell Portal (https://singlecell.broadinstitute.org/single_cell). The dataset includes information on tpm (transcripts per million) values for 6863 malignant cells, 219 oligodendrocytes, 754 macrophages, and 94 T-cells obtained from 28 adult and pediatric patients. For each of the genes encoding MRs, two types of values were calculated separately for four cell types: mean tpm values and percent of cells with non-zero tpm values.

### 2.12 R scripts used in the study

The R scripts implementing all steps of the developed approach are available in the GitHub repository (https://github.com/serivanov86/glioblastoma_MR).

## 3 Results

### 3.1 Transcription factors and upstream master regulators potentially responsible for gene expression changes in GBM

**Fig. 1:**
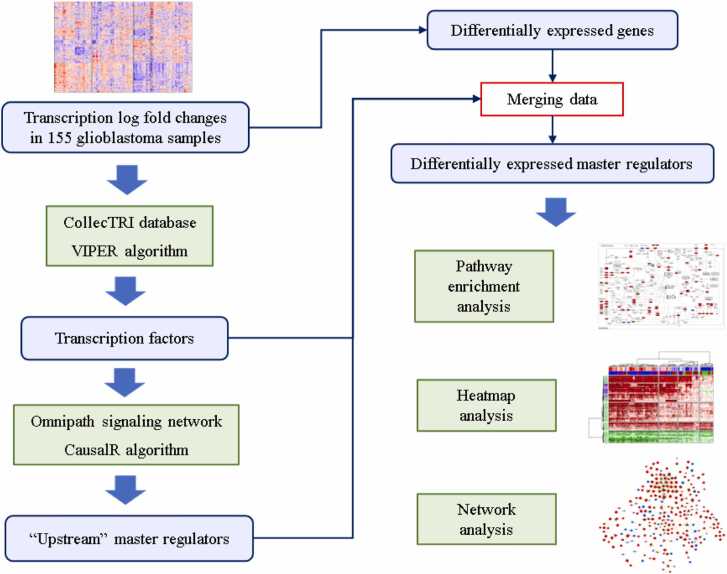
A scheme of the developed workflow aimed at revealing master regulators potentially responsible for gene expression changes in glioblastoma cells.

We developed and applied a workflow (see Materials and Methods) to reveal MRs potentially responsible for gene expression changes in GBM cells (Fig. 1).

**Fig. 2:**
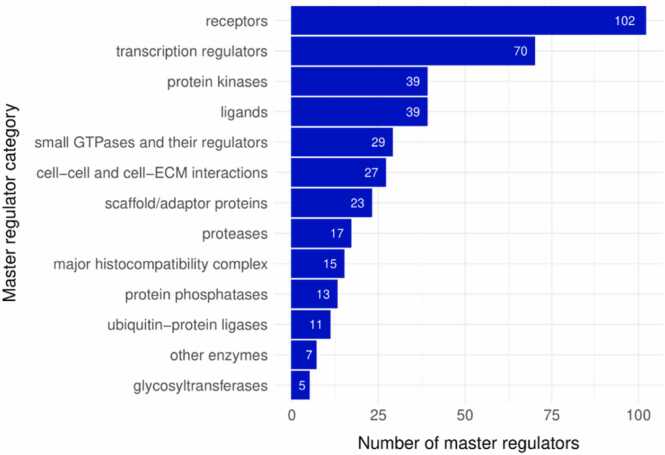
The numbers of master regulators belonging to specific molecular function categories. The figure contains information on 397 out of 451 master regulators. The remaining 54 MRs were classified as “other proteins”.

In the first step, we identified TFs that may directly influence the expression of genes. We revealed 41 “active” and 5 “inactive” TFs (see Table S1). Almost all of them have well-known relationships with GBM pathogenesis, e.g., aryl hydrocarbon receptor (AHR) [55], forkhead box M1 (FOXM1) [56], hypoxia inducible factor 1 subunit alpha (HIF1A) [57], Jun proto-oncogene (JUN) [58], MYC proto-oncogene (MYC) [59], nuclear factor kappa B subunit 1 (NFKB1) [60], signal transducer and activator of transcription 1 and 3 (STAT1, STAT3) [61], [62], etc. The identification of TFs, well studied in GBM, confirms the reliability of the obtained results. In the second step of the analysis, we identified MRs located upstream of TFs in the signaling network (see Materials and Methods). We found 329 “active” and 76 “inactive” MRs (Table S1). Hereinafter, we will refer to both TFs and upstream regulators in the network as MRs. As a result, at both steps of our analysis we identified 451 MRs: 370 “active” and 81 “inactive” ones. Besides TFs, they include various proteins participating in the signaling pathways, including receptors, kinases, small GTPases and their regulators (see Fig. 2). The largest categories were receptors and transcription regulators.

### 3.2 Pathway enrichment analysis

**Fig. 3:**
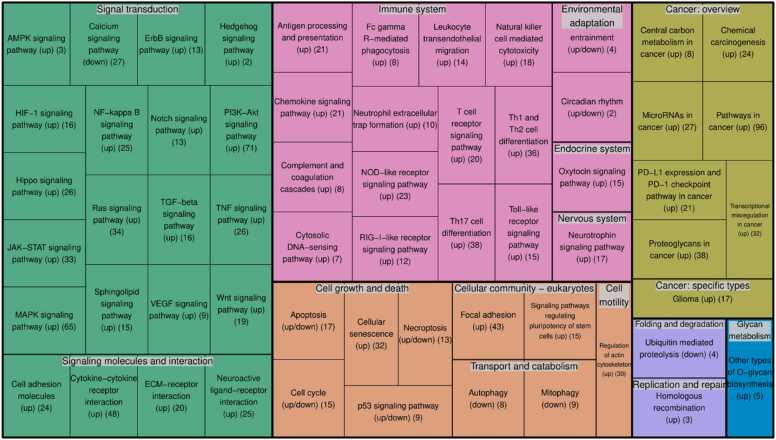
KEGG signaling pathways enriched by the revealed master regulators. Pathways grouped according to the KEGG pathway classification. The data on pathways enrichment by “active” and “inactive” master regulators: “up” or “down”, as well as the numbers of master regulators participated in pathways are shown in brackets.

**Fig. 4:**
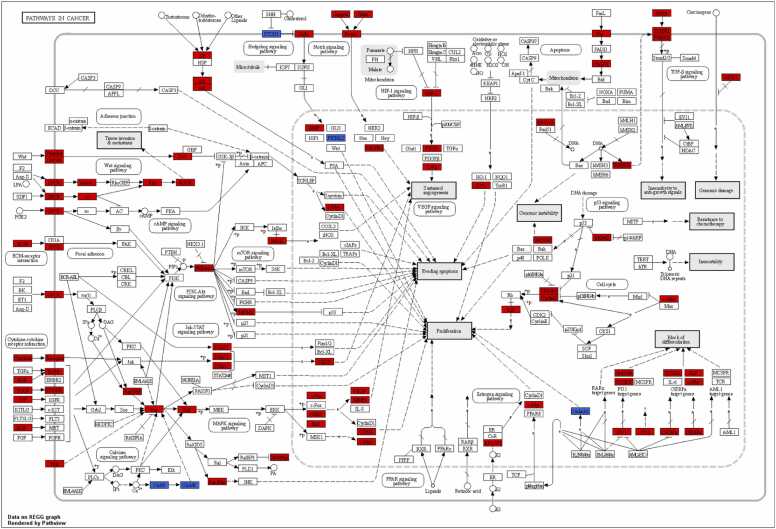
“Pathways in cancer” KEGG map with information on “active” (red color) and “inactive” (blue color) master regulators.

We performed a pathway enrichment analysis to identify the main KEGG pathways containing the revealed MRs (see Materials and Methods) (see Table S2). Fig. 3 contains lists of the obtained pathways grouped according to the KEGG pathway classification. The category with the highest number of enriched pathways is related to a signal transduction in the cell. We identified a significant number of signaling pathways. This is to be expected because MRs are the key components of the signaling network. Most of the identified pathways have an impact on GBM that was confirmed in multiple studies, e.g., developmental pathways such as wnt, hedgehog, and notch [4], [63], [64], [65]. On the other hand, the role of some pathways in GBM biology, including circadian rhythm regulation pathways, oxytocin, and sphingolipid signaling pathways, is less studied. Fig. 4 shows the KEGG map of signaling pathways related to cancer with colored nodes corresponding to the revealed “active” and “inactive” MRs. The identified MRs covered almost all cancer-related pathways regulating cell proliferation, differentiation, apoptosis, and angiogenesis. The map clearly demonstrates cross-talks of signaling pathways that lead to a more complex regulation of cellular processes and the robustness of tumor cells to anti-cancer treatment. The “active” and “inactive” MRs antagonistically regulate downstream proteins.

For example, the receptor PTCH1 that negatively regulates downstream cascades of hedgehog signaling pathway is “inactive”, whereas the positive regulator SMO is “active”. As a result, we concluded that the obtained results on MRs and pathways is consistent and in accordance with the existent knowledge on GBM biology; thus, they can be used in further analysis.

### 3.3 Immune-related master regulators

The second largest category of KEGG pathways is related to both innate and adaptive components of the immune system. We manually classified MRs into immune-specific and non-immune-specific, based on information from the revealed pathways, the UniProt database, and literature. We found that 90 out of 451 MRs can be classified as immune-specific. They include components of the T-cell receptor (e.g., CD4 and CD3E), major histocompatibility complex of class I and II (e.g., HLA-A, HLA-DMA, HLA-DOA, HLA-DQB1, HLA-E, HLA-F), various cytokines and their receptors (e.g., interleukin 2 receptor subunits, interleukin 4, 7 receptors, C-C motif chemokine receptor 5, C-X-C motif chemokine receptor 4, etc.), and pattern recognition receptors (TLR2, TLR4, NOD1, NOD2). Almost all of the immune-specific MRs are “actives”. Pursuant to the “active” state and the known functions of corresponding proteins, we proposed that these MRs are related to a normal immune response, and their discovery may be explained by infiltration of the tumor by immune cells. We found significant correlations between the presence of MRs in individual samples and immune infiltration score (see Materials and Methods) for 75 out of 90 immune-specific MRs (83 %), and for 104 out of 361 non-immune-specific MRs (29 %). Thus, we concluded that the 90 immune-specific MRs may be related to the normal functions of immune cells in the GBM microenvironment rather than to pathological processes in tumor malignant cells.

### 3.4 Distribution of master regulators among tumor samples

**Fig. 5:**
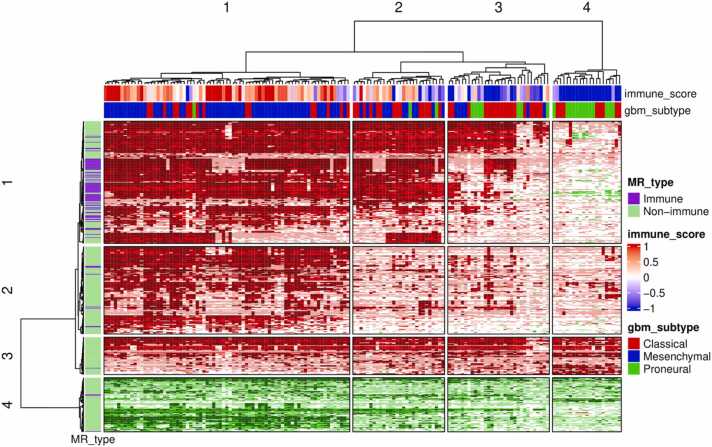
Distribution of master regulators among GBM individual samples. Color shades in the heatmap associate with the merged information on the presence of a master regulator in a particular sample and the expression level of a corresponding encoding gene. If the master regulator was identified as “active” (“inactive”) in a particular GBM sample and the corresponding gene was also up-regulated (down-regulated), it was represented in a dark red (dark green) color. If the master regulator was identified as “active” (“inactive”) in a particular GBM sample but the corresponding gene was not up-regulated (down-regulated), it was represented in red (green) color. If the master regulator was not identified as “active” (“inactive”) in a particular GBM sample but the corresponding gene was up-regulated (down-regulated), it was represented in a light red (green) color. In other cases, the color was considered white. The MR type means that the master regulator is immune-specific or non-immune-specific. The immune score is a normalized immune infiltration score.

To estimate the distribution of the revealed MRs among individual samples, we created a heatmap with hierarchical clustering of samples and proteins (Fig. 5) (see Materials and Methods for details). The heatmap analysis allowed making a number of important observations. First, the clusters of MR profiles generally correspond to GBM transcriptional subtypes. The largest number of MRs were revealed for the MES subtype. Second, samples belonging to the MES subtype in contrast to the CL subtype and, especially, the PN subtypes are associated with significant immune infiltration. The immune-specific MRs are revealed mainly in the MES subtype samples. Third, the numbers of MRs per sample are varied significantly, with the highest number in MES samples and the lowest number in PN samples. Moreover, the MR profiles have a “nested” structure: almost all MRs revealed in PN samples were also revealed in CL samples; however, CL samples contained additional MRs that were absent in PN samples. Similarly, almost all MRs that were revealed in PN and CL samples were also revealed in MES samples; however, MES samples contain additional MRs, which are absent in both PN and CL samples. Fourth, the states of MRs (“active” or “inactive”) are generally the same across the samples where the MR was revealed. Fifth, some MRs (e.g., row cluster 3 in the heatmap) were identified in the most of 155 samples. Among others, many proteins, whose relationships with GBM are well known, were identified in the majority of samples, e.g., epidermal growth factor receptor (EGFR) (129 samples), vascular endothelial growth factor A (VEGFA) (133 samples), hepatocyte growth factor (HGF) (133 samples), angiopoietins 1 and 2 (ANGPT1 and 2) (133 samples), platelet derived growth factor receptor beta (PDGFRB) (129 samples), transforming growth factor beta 1 (TGFB1) (143 samples), forkhead box M1 (FOXM1) (134 samples), signal transducer and activator of transcription 3 (STAT3) (129 samples), matrix metallopeptidase 9 (MMP9) (143 samples), MYC proto-oncogene (MYC) (153 samples), E2F transcription factor 1 (E2F1) (148 samples), and RE1 silencing transcription factor (REST) (all 155 samples).

### 3.5 Analysis of the signaling network containing master regulators

**Fig. 6:**
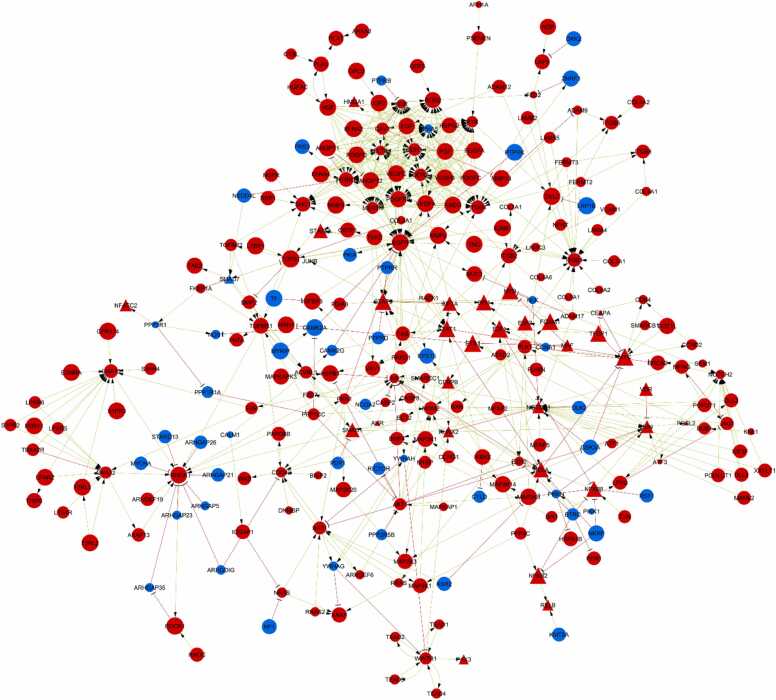
A signaling network consisting of 264 master regulators that directly interact with each other. Triangles indicate transcription factors; ellipses indicate upstream master regulators. Red color indicates “active” master regulators and blue color indicates “inactive” ones. Green arrows represent activating interactions, red arrows represent inhibiting interactions. The size of nodes correlates with the number of samples where the master regulator was identified.

The numbers of the revealed MRs per sample are varied from 18 to 368 with a median of 233. The revealed MRs seem to be not independent but rather interacting, regulating each other and forming a dense interaction network that, in turn, regulates gene expression through TFs. Since the 90 MRs are immune-specific and probably regulate gene transcription in immune cells of an infiltrated tumor, we excluded them from the analysis. We found 359 out of 361 remaining MRs present in the signaling network from OmniPath that contained 3897 proteins expressed in GBM (see Materials and Methods) and 16043 edges of two types: activating and inhibiting. The 264 out of 359 MRs (73.5 %) interacted with each other and formed a single connected subnetwork (Fig. 6). The subnetwork contained 601 interactions, which is twice as many as the number of MRs. It potentially means that the network is very complex and, as a result, resistant to pharmacological perturbation. To demonstrate the significance of the obtained results, we generated 1000 random subnetworks of 359 proteins. The median and maximal sizes of the largest connected components were 79 and 137, whereas the median and maximal numbers of edges were 102 and 218, respectively. It means that the subnetwork of MRs is significantly denser than the random ones.

To estimate the complexity of the MR network and the potential capacity of therapeutic interventions, we performed a controllability analysis (see Materials and Methods). The network is controllable if it can be driven from any initial state to any desired final state within finite time. We used three algorithms to perform the controllability analysis: the minimum driver node set (MDS) [51], the minimum steering node set (MSS) [52], and the steering nodes for output control [53]. The MDS and MSS algorithms were applied to the subnetwork of 264 MRs (Fig. 6) and revealed the same 140 MRs that must be activated to control the “activity” of all 264 MRs in the network. The third algorithm was applied to the whole OmniPath signaling network (3897 proteins and 16043 interactions). It identified 146 out of 359 MRs that must be activated to control the “activity” of all 359 MRs presented in the OmniPath network. These results support the assumption of network complexity, the redundancy of protein functions and robustness of MR network to external pharmacological intervention.

The identified MRs may have different impact on gene expression, GBM development and progression, respectively. We proposed that the most important MRs have central positions in the MR network. We calculated the outdegree and closeness centrality values for each of 264 MRs in the network (Fig. 6) (see Materials and Methods). The higher these values are, the stronger is the influence of a MR on other MRs and gene expression, correspondingly. For instance, the following 17 MRs have output edges to more than 10 other MRs in the network: AKT serine/threonine kinase 1 (AKT1), angiopoietins 1 and 2 (ANGPT1, ANGPT2), amphiregulin (AREG), ephrin A2 (EFNA2), ephrin A4 (EFNA4), epidermal growth factor (EGF), epidermal growth factor receptor (EGFR), hepatocyte growth factor (HGF), insulin like growth factor 2 (IGF2), platelet derived growth factors A, C and D (PDGFA, PDGFC, PDGFD), placental growth factor (PGF), vascular endothelial growth factor A, B and C (VEGFA, VEGFB, VEGFC). The top 20 MRs with the highest closeness centrality additionally include the androgen receptor (AR), protein phosphatase 1 catalytic subunit gamma (PPP1CC), and protein kinase N1 (PKN1). The whole list of 264 MRs interacted with each other along with their outdegree and closeness centrality values is provided in Table S3.

Since some of the identified MRs are TFs which immediately regulate gene transcription, we created a bulk hierarchical TF-target network (see Materials and Methods, Section 2.10). The network contained 42 non-immune TFs and 1320 their target genes differentially expressed in GBM samples, particularly, 182 differentially expressed genes encoding MRs. The two sets of outdegree values in the network were calculated for each TF (Table S4). The first set took into account all DEGs regulated by TFs, the second set took into account only DEGs encoding MRs. The examples of TFs having more than 100 target DEGs included CCAAT enhancer binding protein beta (CEBPB), E2F transcription factor 1 (E2F1), ETS proto-oncogene 1, transcription factor (ETS1), hypoxia inducible factor 1 subunit alpha (HIF1A), Jun proto-oncogene, AP-1 transcription factor subunit (JUN), MYC proto-oncogene, bHLH transcription factor (MYC), nuclear factor kappa B subunit 1 (NFKB1), RELA proto-oncogene, NF-kB subunit (RELA), Spi-1 proto-oncogene (SPI1), signal transducer and activator of transcription 1 and 3 (STAT1, STAT3). These TFs also regulate transcription of 25 to 71 genes encoding MRs; thus, they are components of feedback loops in the gene expression regulatory network and may be viral for GBM development and progression. The whole list of 42 TFs along with their outdegree values is provided in Table S4.

### 3.6 Validation of expression of master regulator - encoding genes using single-cell RNA sequencing data

The chosen single-cell RNA sequencing dataset includes information on tpm (transcripts per million) values for 6863 malignant cells, 219 oligodendrocytes, 754 macrophages, and 94 T-cells (see Materials and Methods). The tpm values were available for 444 out of 451 genes encoding MRs: 90 immune-related genes and 354 tumor-related genes. For each of 444 genes and each of four cell types, two values were calculated: a mean tpm value and percent of cells with non-zero tpm values (see Table S5).

**Fig. 7:**
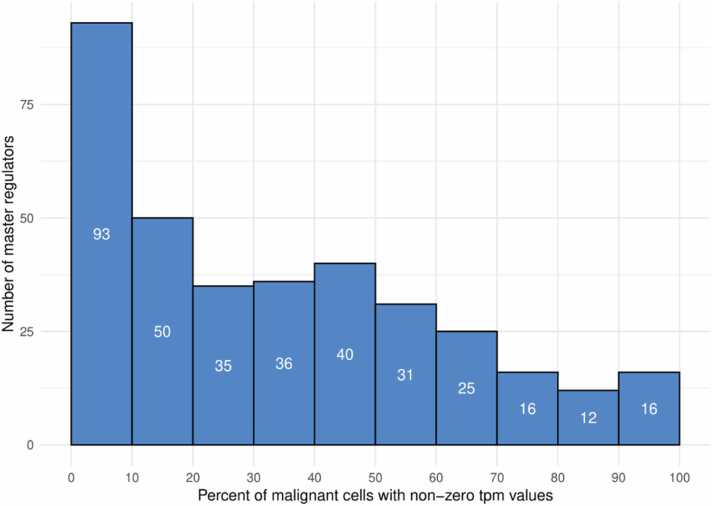
The numbers of 354 tumor-related master regulators having non-zero tpm values in different percentages of malignant cells.

We found that the average tpm for immune-related genes was 8 times more than for tumor-related genes in macrophages and 5 times more in T-cells. On the contrary, the average tpm for immune-related genes was 1.5 times less than for tumor-related genes in malignant cells. This is in accordance with the hypothesis that 90 MRs are related to immune cells in the tumor microenvironment rather than to malignant cells. All 354 tumor-related genes have non-zero average tpm values in malignant cells, but the percent of malignant cells with non-zero tpm is different for particular genes (see Fig. 7). For instance, 93 genes are expressed in less than 10 % of malignant cells, whereas 16 genes (AAK1, BTF3, CALM1, CASP2, CDC42, DDR1, GSTP1, HMGN1, ITGB8, MAP1B, MDM2, PABPC1, RAN, RHOA, S1PR2, TMED10) are expressed in more than 90 % of cells. Generally, the expression of most of 354 MRs in malignant cells is confirmed at a single cell level.

### 3.7 Particular sets of master regulators

Despite the fact that gene transcription in GBM seems to be regulated by a complex network of MRs rather than by the individual ones, the corresponding network has a modular structure (see Figs. 4 and 6), where each module corresponds to a known signaling pathway (Fig. 3). To demonstrate the role of the revealed pathways in GBM, we selected MRs, which are the key nodes of corresponding pathways, to analyze them in detail (Table 1). Table 1 contains the information of MRs participating in pathways revealed by enrichment analysis (Fig. 3).

**Table 1:** Important master regulators from signaling pathways that are essential for glioblastoma and are identified by enrichment analysis.

**Gene**	**Protein name**	**State**^a^	**Pathway**	**N of samples**^b^	**N MES**^b^	**N CL**^b^	**N PN**^b^
GPR161	G protein-coupled receptor 161	A	Hedgehog signaling pathway	61	18 (23.7)	31 (55.4)	12 (52.2)
PTCH1	patched 1	I		25	20 (26.3)	5 (8.9)	0 (0)
SMO	smoothened, frizzled class receptor	A		86	60 (78.9)	24 (42.9)	2 (8.7)
TEAD1	TEA domain transcription factor 1	A	Hippo signaling pathway	26	8 (10.5)	15 (26.8)	3 (13)
TEAD2	TEA domain transcription factor 2	A		30	12 (15.8)	15 (26.8)	3 (13)
TEAD3	TEA domain transcription factor 3	A		32	13 (17.1)	17 (30.4)	2 (8.7)
TEAD4	TEA domain transcription factor 4	A		16	7 (9.2)	8 (14.3)	1 (4.3)
WWTR1	WW domain containing transcription regulator 1	A		49	17 (22.4)	22 (39.3)	10 (43.5)
DLK2	delta like non-canonical Notch ligand 2	I	Notch signaling pathway	60	40 (52.6)	19 (33.9)	1 (4.3)
DLL3	delta like canonical Notch ligand 3	A		48	31 (40.8)	15 (26.8)	2 (8.7)
DLL4	delta like canonical Notch ligand 4	A		24	18 (23.7)	6 (10.7)	0 (0)
JAG1	jagged canonical Notch ligand 1	A		61	41 (53.9)	18 (32.1)	2 (8.7)
NOTCH1	notch receptor 1	A		34	11 (14.5)	23 (41.1)	0 (0)
NOTCH2	notch receptor 2	A		22	16 (21.1)	6 (10.7)	0 (0)
BMP2	bone morphogenetic protein 2	A	TGF-beta signaling pathway	23	14 (18.4)	4 (7.1)	5 (21.7)
BMP4	bone morphogenetic protein 4	A		26	16 (21.1)	5 (8.9)	5 (21.7)
GDF5	growth differentiation factor 5	A		24	19 (25)	4 (7.1)	1 (4.3)
INHBB	inhibin subunit beta B	A		36	20 (26.3)	14 (25)	2 (8.7)
LEFTY2	left-right determination factor 2	A		65	43 (56.6)	14 (25)	8 (34.8)
NODAL	nodal growth differentiation factor	A		58	35 (46.1)	14 (25)	9 (39.1)
SMAD1	SMAD family member 1	A		55	36 (47.4)	14 (25)	5 (21.7)
SMAD5	SMAD family member 5	A		28	21 (27.6)	6 (10.7)	1 (4.3)
SMAD7	SMAD family member 7	I		26	19 (25)	5 (8.9)	2 (8.7)
TGFB1	transforming growth factor beta 1	A		108	65 (85.5)	32 (57.1)	11 (47.8)
TGFBR1	transforming growth factor beta receptor 1	A		83	56 (73.7)	15 (26.8)	12 (52.2)
TGFBR2	transforming growth factor beta receptor 2	A		35	29 (38.2)	6 (10.7)	0 (0)
CXXC4	CXXC finger protein 4	I	Wnt signaling pathway	11	8 (10.5)	3 (5.4)	0 (0)
DKK2	dickkopf WNT signaling pathway inhibitor 2	I		66	23 (30.3)	24 (42.9)	19 (82.6)
DVL2	dishevelled segment polarity protein 2	A		31	10 (13.2)	17 (30.4)	4 (17.4)
FZD2	frizzled class receptor 2	A		16	7 (9.2)	6 (10.7)	3 (13)
FZD7	frizzled class receptor 7	A		18	13 (17.1)	5 (8.9)	0 (0)
LRP1B	LDL receptor related protein 1B	I		105	60 (78.9)	32 (57.1)	13 (56.5)
LRP5	LDL receptor related protein 5	A		71	21 (27.6)	33 (58.9)	17 (73.9)
ROR2	receptor tyrosine kinase like orphan receptor 2	A		27	23 (30.3)	1 (1.8)	3 (13)
RYK	receptor like tyrosine kinase	A		35	25 (32.9)	8 (14.3)	2 (8.7)
CALM1	calmodulin 1	I	Calcium signaling pathway	15	0 (0)	3 (5.4)	12 (52.2)
CAMK2A	calcium/calmodulin dependent protein kinase II alpha	I		128	69 (90.8)	43 (76.8)	16 (69.6)
CAMK2G	calcium/calmodulin dependent protein kinase II gamma	I		32	9 (11.8)	10 (17.9)	13 (56.5)
NOS1	nitric oxide synthase 1	I		27	13 (17.1)	10 (17.9)	4 (17.4)
CLOCK	clock circadian regulator	I	Circadian rhythm	7	2 (2.6)	0 (0)	5 (21.7)
PER1	period circadian regulator 1	A		26	17 (22.4)	7 (12.5)	2 (8.7)
PRKAG1	protein kinase AMP-activated non-catalytic subunit gamma 1	A	AMPK signaling pathway	28	18 (23.7)	9 (16.1)	1 (4.3)

a A is “active” master regulator, I is “inactive” master regulator.

b N of samples is the number of samples where the master regulator was identified as differentially expressed; N MES, N CL, N PN are the corresponding numbers calculated for glioblastoma subtypes. The percent of samples belonging to particular glioblastoma subtypes, where the master regulator was identified as differentially expressed, is given in brackets.

Since 102 out of 451 MRs (23 %) were classified as receptors, we selected 25 of them with little or no information regarding their role in GBM (Table 2). They were further grouped by type of their ligands, e.g., nucleotide-like receptors, fatty acid receptors, lysophospholipid receptors, eicosanoid receptors. The potential role of key proteins from enriched pathways (Table 1) and receptors (Table 2) in GBM development and progression is mentioned in the Discussion section.

**Table 2:** Receptors identified as master regulators with little or no studied role in glioblastoma.

**Gene**	**Protein name**	**Ligands**	**N of samples**^a^	**N MES**^a^	**N CL**^a^	**N PN**^a^
**Nucleotide-like receptors**						
ADORA3	adenosine A3 receptor	adenosine	50	33 (43.4)	14 (25)	3 (13)
P2RY1	purinergic receptor P2Y1	adenine nucleotides, e.g., ADP	80	45 (59.2)	35 (62.5)	0 (0)
**Thrombin receptors**						
F2R	coagulation factor II thrombin receptor	activated thrombin	53	33 (43.4)	17 (30.4)	3 (13)
F2RL2	coagulation factor II thrombin receptor like 2	activated thrombin	122	75 (98.7)	42 (75)	5 (21.7)
F2RL3	F2R like thrombin or trypsin receptor 3	activated thrombin	118	67 (88.2)	43 (76.8)	8 (34.8)
**Fatty acid receptors**						
GPR132	G protein-coupled receptor 132	oxidized free fatty acids such as 9-hydroxyoctadecadienoic acid	33	31 (40.8)	2 (3.6)	0 (0)
GPR84	G protein-coupled receptor 84	fatty acids: capric acid, undecanoic acid and lauric acid	35	25 (32.9)	10 (17.9)	0 (0)
**Lysophospholipid receptors**						
GPR174	G protein-coupled receptor 174	lysophosphatidylserine	48	30 (39.5)	11 (19.6)	7 (30.4)
GPR34	G protein-coupled receptor 34	lysophosphatidylserine	44	30 (39.5)	11 (19.6)	3 (13)
LPAR2	lysophosphatidic acid receptor 2	lysophosphatidic acid	45	24 (31.6)	16 (28.6)	5 (21.7)
LPAR5	lysophosphatidic acid receptor 5	lysophosphatidic acid	22	16 (21.1)	5 (8.9)	1 (4.3)
LPAR6	lysophosphatidic acid receptor 6	lysophosphatidic acid	46	30 (39.5)	14 (25)	2 (8.7)
S1PR2	sphingosine−1-phosphate receptor 2	sphingosine 1-phosphate	35	19 (25)	14 (25)	2 (8.7)
S1PR3	sphingosine−1-phosphate receptor 3	sphingosine 1-phosphate	125	75 (98.7)	43 (76.8)	7 (30.4)
S1PR4	sphingosine−1-phosphate receptor 4	sphingosine 1-phosphate	25	19 (25)	5 (8.9)	1 (4.3)
PTAFR	platelet activating factor receptor	platelet activating factor	62	48 (63.2)	12 (21.4)	2 (8.7)
**Eicosanoid receptors**						
CYSLTR1	cysteinyl leukotriene receptor 1	leukotrienes: LTD4 > > LTE4 = LTC4 > > LTB4	26	19 (25)	7 (12.5)	0 (0)
LTB4R	leukotriene B4 receptor	leukotriene B4, extracellular ATP > UTP and ADP	28	18 (23.7)	9 (16.1)	1 (4.3)
PTGER4	prostaglandin E receptor 4	prostaglandin E2	41	36 (47.4)	4 (7.1)	1 (4.3)
PTGIR	prostaglandin I2 receptor	prostacyclin	52	27 (35.5)	21 (37.5)	4 (17.4)
TBXA2R	thromboxane A2 receptor	thromboxane A2	47	26 (34.2)	20 (35.7)	1 (4.3)
**Other receptors**						
GALR1	galanin receptor 1	galanin	28	16 (21.1)	10 (17.9)	2 (8.7)
HRH1	histamine receptor H1	histamine	51	34 (44.7)	16 (28.6)	1 (4.3)
MC1R	melanocortin 1 receptor	melanocyte-stimulating hormone, adrenocorticotropic hormone	43	18 (23.7)	19 (33.9)	6 (26.1)
OXTR	oxytocin receptor	oxytocin	38	24 (31.6)	14 (25)	0 (0)

a N of samples is the number of samples where the master regulator was identified as differentially expressed; N MES, N CL, N PN are the corresponding numbers calculated for glioblastoma subtypes. The percent of samples belonging to particular glioblastoma subtypes, where the master regulator was identified as differentially expressed, is given in brackets.

## 4 DISCUSSION

We have developed a network-based approach aimed at identifying MRs, which are the proteins in the signaling network responsible for the induction and maintenance of gene expression changes in GBM cells compared to healthy tissues. The main feature of our approach is that all calculations are performed for transcription profiles from individual samples with subsequent estimation of the MR distribution between them, calculation of correlations between the MR presence in the samples and corresponding changes in the transcription of encoding genes, and immune infiltration scores. This approach allowed identifying both common and rare MRs revealed in small numbers of samples. The application of the approach allowed us to reveal MRs important for GBM development and progression and estimate their properties, including interactions between MRs in the signaling network. We believe that the results of our approach can be of high relevance for the development of individual strategies for GBM treatment and cure.

We identified MRs of two types: (1) TFs responsible for the observed transcription changes in GBM compared to healthy tissues, and (2) “upstream” MRs, which regulate activities of TFs through interactions in the signaling network. On the other hand, MRs were subdivided into “actives” and “inactives”. “Active” MRs were predicted by network analysis to stimulate expression of genes that were found up-regulated in GBM (see Materials and Methods) and repress expression of genes that were found down-regulated. “Inactive” MRs were predicted to repress expression of genes that were found up-regulated in GBM and stimulate expression of genes that were found down-regulated. The pharmacological stimulation of “inactive” MRs and/or inhibition of “active” MRs would reverse gene expression profiles to those in healthy tissues and demonstrate an anti-tumor effect. This idea was realized in the “Connectivity Map” approach [66], [67] that was verified for many tumor- and non-tumor diseases, including GBM [68], [69], [70], [71], in in vitro and in vivo studies.

We supposed that MRs that have the highest significance for GBM development must regulate their own expression differentially: genes encoding “active” MRs should be up-regulated, whereas genes encoding “inactive” MRs should be down-regulated. In such cases, MRs are the parts of positive feedback loops that have an important role in cancer [72]. We identified 451 such MRs: 370 “active” and 81 “inactive” ones with a high number of receptors among them (Fig. 2).

The distribution of the revealed MRs among individual samples was estimated by creating the heatmap with a hierarchical clustering of samples and proteins. We have made some observations regarding the MR distribution (see Section 3.4 of the Results). Importantly, we found that the clusters of MR profiles generally correspond to GBM transcriptional subtypes, with the highest number of MRs in MES samples and the lowest number in PN samples. The deconvolution analysis also revealed that the samples belonging to MES, and partially the CL subtype, are associated with significant immune infiltration. It is known that MES tumors are significantly infiltrated by various immune cells compared to other subtypes [73]; however, the functions of immune cells in the tumor microenvironment are suppressed. Feng Q. and colleagues demonstrated that the increased immune infiltration of gliomas with the altered functions of immune cells was associated with a higher tumor stemness, epithelial-mesenchymal transition scores, and genomic instability that leads to poor survival prognosis [74]. We identified 90 immune-related MRs that were associated with GBM samples having high immune infiltration scores (Fig. 5). The majority of them seems to be associated with normal functioning of immune cells, e.g., T-cell receptor components (CD4 and CD3E), major histocompatibility complex of class I and II (e HLA-A, HLA-DMA, HLA-DOA, HLA-DQB1, HLA-E, HLA-F), various cytokines and their receptors, whereas some of them are clearly associated with immune suppression, e.g., interleukin 10 receptor (IL10RA), and receptor for interleukins 4 and 13 [75], [76]. Several recent studies showed that the high expression of various gene encoding immune-related MRs that are known to be important components of anti-tumor immune response, e.g., interferon gamma receptor (IFNGR2) [77], [78], [79], and pattern recognition receptors NOD1 and NOD2 [80], was associated with GBM progression and lower survival. These findings can be explained by the fact that these molecules are expressed on various immune and non-immune cells in the GBM core and peritumoral microenvironment, which, in turn, form a complex cell-cell interaction network. The real phenotype of a tumor is a result of simultaneous interactions between molecules and cells rather than a result of action of particular cytokines and growth factors [81], [82].

Another important observation in our study is that the MR profiles have a “nested” structure: almost all MRs revealed in PN samples were also revealed in CL samples; however, CL samples contained additional MRs that were absent in PN samples. Similarly, almost all MRs revealed in PN and CL samples were also revealed in MES samples; however, MES samples contain additional MRs that were absent in both PN and CL samples. We proposed that GBM subtypes generally are not characterized by their own distinct profiles of MRs and that the differences between them are more “quantitative” than “qualitative”. These findings are in line with the known high plasticity of subtypes [18], [83], [84]. The single cell transcriptomics analysis revealed four main cellular states of malignant cells in GBM: NPC-like, OPC-like, AC-like, and MES-like. The GBM subtypes observed at the level of transcription profiles obtained by bulk RNA sequencing are defined by the proportions of these types of cells: CL and MES subtypes correspond to tumors enriched for the AC-like and MES-like states, respectively, and the PN subtype corresponds to the combination of two distinct cellular states, OPC-like and NPC-like [18]. The corresponding proportions are varied between different regions of the same tumor, and, in some samples, the proportions of cell types are intermediate, and the corresponding sample cannot be clearly classified to one of three GBM subtypes [15]. It is currently known that hybrid cells, which have properties of several cell types: NPC-like, OPC-like, AC-like, and MES-like, may also occur. The cell type plasticity means that a cell of one type is able to transit to another type, and, in turn, one GBM subtype is able to transit to another subtype. The most common type of such transition is proneural-mesenchymal transition [83]. Importantly, some of the revealed MRs are well-known regulators of such transitions. For example, neurofibromin 1 (NF1) was revealed as “inactive” MR mainly in samples of the MES subtype (see Table S1). It is known that inactivation of NF1 due mutations is the main driver of acquiring mesenchymal phenotype [18], [85]. NF1 deficiency also resulted in an increased tumor-associated macrophages/microglia infiltration. The resulted high immune infiltration was, in turn, associated with the epithelial-mesenchymal transition. Thus, intrinsic and microenvironmental features of the GBM ecosystem may influence each other and define the plasticity and intra-tumoral heterogeneity.

Fig. 5 demonstrates that the MES subtype is associated both with the highest numbers of MRs and high immune infiltration. It can be proposed that the more complex the tumor microenvironment, including a higher number of cell types, the higher number of MRs involved in gene expression regulation are required.

The relationships of the identified MRs with malignant and immune cells were confirmed at a single-cell level. We found that 90 immune-related MRs have significantly higher expression in immune cells (macrophages and T-cells) than in malignant cells (see Results, Section 3.6). On the contrary, other 354 MRs have significantly higher expression levels in malignant cells. All genes encoding 354 MRs have non-zero average tpm values in malignant cells that confirm their expression at the single-cell level. The percent of malignant cells with non-zero tpm is different for particular genes (see Fig. 7), which reflects the GBM cell heterogeneity. The 16 genes encoding the following MRs: AAK1, BTF3, CALM1, CASP2, CDC42, DDR1, GSTP1, HMGN1, ITGB8, MAP1B, MDM2, PABPC1, RAN, RHOA, S1PR2, TMED10) were expressed in more than 90 % of cells. These genes may present interest for further research since their wide expression in the malignant cells may reflect their important roles in the GBM progression.

The revealed MRs are not independent regulators but they interact and regulate each other and form a dense interaction network that, in turn, regulates gene expression through TFs. We found that 264 out of 359 (73.5 %) non-immune MRs interacted with each other in the signaling network and formed a single connected subnetwork with a high number of edges. It potentially means that the network is very complex and, as a result, resistant to pharmacological perturbation. This network can be represented as a superposition of 155 networks regulating gene expression in individual GBM samples. In other words, gene expression in each of 155 GBM samples is regulated by their own networks of MRs and merging these 155 networks into one will result in the network of 264 non-immune MRs (see Fig. 6). The numbers of the revealed MRs per sample are varied from 18 to 368 so that the corresponding networks are also varied in the MR content and complexity. We proposed that pharmacological therapy should directly or indirectly influence all MRs in the merged network to achieve a desirable anti-tumor effect in all 155 GBM samples. Controllability analysis showed that it is necessary to perturb 140 MRs to control the “activity” of all 264 non-immune MRs. These results support the assumption of the network complexity, redundancy of protein functions, and robustness of the MR network to external pharmacological intervention. Recently, Arash Sadri proposed that the target-based drug discovery has been inefficient in the creation of new drugs for the treatment of complex diseases such as GBM. It was shown that approved drugs mediate their therapeutic effects through numerous off-target mechanisms rather than a single target [86]. Thus, to achieve the desired anti-tumor effect, multi-target drug action or the application of synergistic drug combinations are required. Kinase inhibitors have multiple off-targets that make them attractive candidates for targeted therapy of GBM [87]. Importantly, the kinase inhibitors that are potentially applicable for GBM treatment are known to inhibit many of the revealed MRs [5], [87], [88], [89]. Unfortunately, to date, most of the corresponding clinical trials have failed due to the insufficient efficacy of corresponding inhibitors. The possible reasons are the low permeability of blood-brain and blood-tumor barriers for these drugs and the complexity of regulatory networks. Thus, to achieve the anti-tumor effect, both the selection of synergistic drug combination [90], [91], [92] and the development of drug delivery systems [93], [94] are potentially required.

On the contrary, the controllability analysis may overestimate the number of potential pharmacological targets, which must be perturbed simultaneously to achieve the anti-tumor effect. This is due to the non-equal importance of MRs in the induction of gene expression. We proposed that the most important MRs have central position in the network of 264 MRs that can be determined by calculation of outdegree and closeness centrality values (see Materials and Methods and Results sections). The higher these values, the stronger the influence of MR on other MRs and gene expression, correspondingly. MRs with the highest outdegree and closeness centrality values include those identified in most of 155 GBM samples, e.g., amphiregulin (AREG) (133 samples), angiopoietins 1 and 2 (ANGPT1 and 2) (133 samples), ephrins A2 and A4 (EFNA2 and 4) (133 samples), epidermal growth factor receptor (EGFR) (129 samples), hepatocyte growth factor (HGF) (133 samples), insulin like growth factor 2 (IGF2) (133 samples), platelet derived growth factors A, C and D (PDGFA, PDGFC, PDGFD) (133 samples), placental growth factor (PGF) (133 samples), vascular endothelial growth factor A, B and C (VEGFA, VEGFB, VEGFC) (133 samples). The central position in the network and presence in most of GBM samples potentially suggest these MRs to be the most important for GBM progression and development. The relationships of the revealed MRs with GBM are confirmed by multiple studies. For instance, vascular endothelial growth factors, angiopoietins, and placental growth factor play an important role in GBM angiogenesis [95], [96]. Epidermal growth factor receptor (EGFR) is one of the most important and well-studied regulators of GBM progression, whereas amphiregulin (AREG) is one of its ligands. Platelet-derived growth factors determine the GBM subtype [15] and promote angiogenesis [97]. Ephrins A2 and A4 are tumorigenic molecules enhancing proliferation, migration, and de-differentiation of GBM cells [98], [99]. The role of hepatocyte growth factor and insulin-like growth factor 2 in GBM was also described in the literature [100], [101].

Additionally, through a similar analysis of the bulk hierarchical TF-target network, we identified TFs that potentially have the strongest influence on gene expression in GBM (see Materials and Methods and Results sections). Using the calculation of outdegree values, we identified TFs regulating the expression of the high number of DEGs and, particularly, genes encoding MRs. Particularly, we identified 11 TFs regulating the transcription of more than 100 target DEGs and more than 25 DEGs encoding MRs. Thus, these TFs are the key components of feedback loops in the gene expression regulatory network and may be viral for the GBM development and progression. Most of them have well-known relationships with GBM pathogenesis, e.g., hypoxia inducible factor 1 subunit alpha (HIF1A) [57], Jun proto-oncogene, AP-1 transcription factor subunit (JUN) [58], MYC proto-oncogene, bHLH transcription factor (MYC) [59], nuclear factor kappa B subunit 1 (NFKB1) [60], signal transducer, and activator of transcription 1 and 3 (STAT1, STAT3) [61], [62].

Despite the revealed MRs densely interacting with each other, the corresponding network has a modular structure (Fig. 6). The modules generally correspond to particular signaling pathways. Pathway enrichment analysis revealed several signaling pathways with important roles in GBM development and progression (Fig. 3). Table 1 contains data on key proteins of the selected signaling pathways, which were identified as MRs. Most of the MRs in Table 1 belong to major signaling pathways involved in the developmental process, such as Notch, Hedgehog, Wnt, and TGF-beta. The role of these pathways in GBM development, progression, patient survival and resistance to treatment as well as the role of mutations and epigenetic regulation in their up-regulation in GBM are well described in the literature [4], [63], [64], [65]. For instance, up-regulation of the Hedgehog pathway is associated with GBM proliferation and progression by enhancing cancer stem cells. The functional activity of pathway components can be changed by both activating or inhibiting mutations and epigenetic mechanisms [4], [63]. We found that the two most important components of the Hedgehog pathway, PTCH1 and SMO, were mainly associated with samples of the MES GBM subtype (see Table 1). It was shown that inhibition of the Hedgehog pathway prevented epithelial-mesenchymal transition [102]; however, the effect was stronger if additional pathways, e.g., TGF-beta, Wnt, AMPK, or PI3K/Akt/mTOR, were blocked [103], [104]. This observation is in agreement with the redundancy of MR functions and complexity of the MR interaction network.

It is currently known that cross-talk signals between cancer and immune cells through ligand-receptors interactions in the tumor are especially important for GBM evolution [105], [106]. We identified 102 receptors (23 %) among 451 revealed MRs, which is the largest molecular function category (see Fig. 2). The role of some of the revealed receptors in GBM, e.g., EGFR, NOTCH1 and 2, PDGFRB, is well described in the literature, whereas other receptors were less or not studied. We selected 25 receptors with little or no information regarding their role in GBM (see Table 2). We found that they belong to the same groups of ligands and, thus, can be grouped into nucleotide-like receptors, thrombin receptors, fatty acid receptors, lysophospholipid receptors, and eicosanoid receptors. The largest group contains various lysophospholipid receptors, which, in turn, are related to three ligands: lysophosphatidylserine, lysophosphatidic acid, and sphingosine 1-phosphate. The role of lysophosphatidic acid and sphingosine 1-phosphate in GBM is well described [107], [108]; however, the impact of particular receptors, e.g., LPAR5, LPAR6, and S1PR4, is not currently known. The information about lysophosphatidylserine and its receptors is also limited. It is only known that the stimulation of GPR34 enhances the malignancy and carcinogenesis of glioma by promoting an epithelial-mesenchymal transition, G1/S phase cell cycle transition, and TGF-beta/Smad signaling [109]. The role in GBM for GPR84, which is a receptor for capric, undecanoic, and lauric fatty acids, and GPR132, which is a receptor for oxidized free fatty acids such as 9-hydroxyoctadecadienoic acid, is also not studied. We proposed that the obtained information on the potential role of various receptors listed in Table 2 in GBM may be a basis for further research.

## 5 Conclusion

We have developed an approach for the identification of master regulators that are responsible for the gene expression changes in glioblastoma cells compared to healthy tissues. The main feature of the approach is that all calculations are performed for transcription profiles from individual samples, which allowed revealing common master regulators associated with most of samples and rare master regulators associated with small number of samples. The analysis of 451 revealed master regulators allowed making the following conclusions: (1) the number of master regulators increases with an increase in the complexity of the microenvironment and immune infiltration, and the highest number was associated with the mesenchymal subtype of glioblastoma, whereas the lowest number was associated with the proneural subtype of glioblastoma; (2) generally, the glioblastoma subtypes are not characterized by their own distinct profiles of master regulators, and the differences between them are more “quantitative” than “qualitative”, which is in agreement with the known high subtype plasticity; (3) the revealed master regulators interact with each other in the signaling network and form a dense subnetwork that is associated with a potential robustness to pharmacological perturbation. To achieve the anti-tumor effect in all patients, a synergistic drug combination coupled with drug delivery systems is potentially required; (4) the largest class of master regulators are receptors, which is in agreement with the importance of cell-cell interactions for glioblastoma development and progression. We found several new receptors whose role in glioblastoma is not currently investigated. We believe that the developed approach can be used to search for novel therapeutic strategies to treat GBM as well as to identify important regulators of gene expression in other tumors.

## Funding

This work was financed by the 10.13039/501100012190Ministry of Science and Higher Education of the Russian Federation within the framework of state support for the creation and development of World-Class Research Centers "Digital Biodesign and Personalized Healthcare" (No. 075-15-2022-305).

## Declaration of Competing Interest

none.

## Footnotes

Appendix A Supplementary data associated with this article can be found in the online version at doi:10.1016/j.csbj.2024.09.022.
